# Steep repolarization time gradients in pig hearts cause distinct changes in composite electrocardiographic T‐wave parameters

**DOI:** 10.1111/anec.12994

**Published:** 2022-08-19

**Authors:** Jeanne van der Waal, Laura Bear, Veronique Meijborg, Rémi Dubois, Matthijs Cluitmans, Ruben Coronel

**Affiliations:** ^1^ Department of Experimental and Clinical Cardiology Amsterdam UMC, Location AMC Amsterdam The Netherlands; ^2^ IHU Liryc, Electrophysiology and Heart Modeling Institute Fondation Bordeaux Université Pessac France; ^3^ Université de Bordeaux Pessac France; ^4^ Inserm, Cardio‐Thoracix Research Centre of Bordeaux Pessac France; ^5^ CARIM School for Cardiovascular Diseases Maastricht University Medical Centre Maastricht The Netherlands

**Keywords:** 12‐lead ECG, cardiac repolarization, electrophysiology, repolarization heterogeneity, torso tank, T_peak–end_/Tpe

## Abstract

**Background:**

The T wave of the electrocardiogram (ECG) reflects ventricular repolarization. Repolarization heterogeneity is associated with reentrant arrhythmias. Several T‐wave markers (including QT interval) have been associated with ventricular arrhythmias, but studies linking such markers to underlying local repolarization time (RT) inhomogeneities are lacking. We aimed to investigate the relation of several T‐wave markers to controlled drug‐induced regional RT gradients in intact pig hearts.

**Methods:**

Repolarization time gradients were created by regional infusion of dofetilide and pinacidil in four atrially paced porcine Langendorff‐perfused hearts placed inside a torso tank. From the 12‐lead ECG on the torso tank, the mean, maximum, and dispersion (max–min) of QT_time_, JT_time_, T_peak–end_, T_width_, TQ_ratio_, dV/dt_max_, T_area_, T_amp_, and T‐upslope duration were determined, as well as upslope end difference between leads V_1_ and V_6_.

**Results:**

Temporal T‐wave parameters T_peak–end_, T_width,_ and TQ_ratio_ show a significant and high correlation with RT gradient, best reflected by mean value. T_area_ (mean, max and dispersion) and dV/dt_max_ dispersion show only a moderate significant correlation. T‐upslope duration shows a significant correlation in particular for mean values. Mean, maximum, or dispersion of QT_time_ and V_1_–V_6_ upslope end difference were not significantly correlated with RT gradient.

**Conclusion:**

Composite 12‐lead ECG T‐wave parameters T_peak–end_, T_width_, TQ_ratio_, upslope duration, and T_area_ show a good correlation with underlying RT heterogeneity, whereas standard clinical metrics such as QT_time_ do not reflect local RT heterogeneity. The composite T‐wave metrics may thus provide better insights in arrhythmia susceptibility than traditional QT_time_ metrics.

## Graphical abstract

In this article, we investigated the relation between (drug‐induced) repolarization time gradients (top) and T‐wave markers based on the 12‐lead ECG (bottom). We found that composite T‐wave markers, such as T_peak–end_, T_width,_ and T_area,_ show a good correlation with underlying RT heterogeneity, whereas standard clinical metrics such as QT_time_ do not reflect local RT heterogeneity.

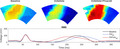

## 1 INTRODUCTION

Cardiac ventricular arrhythmias are an important cause of death (Montagnana et al., 2008), and they are commonly caused by reentrant activation. Increased local repolarization gradients facilitate the initiation of reentry and life‐threatening arrhythmias (Han & Moe, 1964; Rivaud et al., 2021). Early risk stratification of patients with increased repolarization heterogeneities may help to reduce mortality caused by arrhythmias. The electrocardiogram (ECG) is a widely used clinical screening tool that may provide such information.

The QT interval as a traditional arrhythmia risk marker reflects repolarization duration rather than local repolarization time (RT) gradients. The few studies directly relating T‐wave parameters with underlying repolarization generally describe global rather than local repolarization inhomogeneities reflected as RT gradient (Srinivasan et al., 2019). Non‐invasive ECG markers that reflect actual underlying local RT inhomogeneities and not global repolarization duration may provide more relevant information about arrhythmic substrates and could be used to aid stratification of patients at risk for SCD. Although several T‐wave markers (e.g., the (heart rate corrected) QT interval [QT(c)], the JT interval, the T_peak_–T_end_ [TpTe] interval) have been associated with sudden cardiac death (SCD) or ventricular arrhythmias (Tse et al., 2018), the exact relation between the T‐wave morphology and underlying repolarization heterogeneity is not understood.

Here, we used selective intracoronary drug infusion to create several degrees of local RT inhomogeneities in intact pig hearts. We performed simultaneous measurement of body‐surface ECG and epicardial RT patterns. We studied the relation of several electrocardiographic T‐wave markers with the underlying local RT gradients.

## 2 METHODS

All experiments were approved by the institutional review committee for experiments on animals, and animal handling was in accordance with the European Directive for the Protection of Vertebrate Animals Used for Experimental and Other Scientific Purposes (European Union Directive 86/609/EEC).

### 2.1 Experimental protocol

**FIGURE 1:**
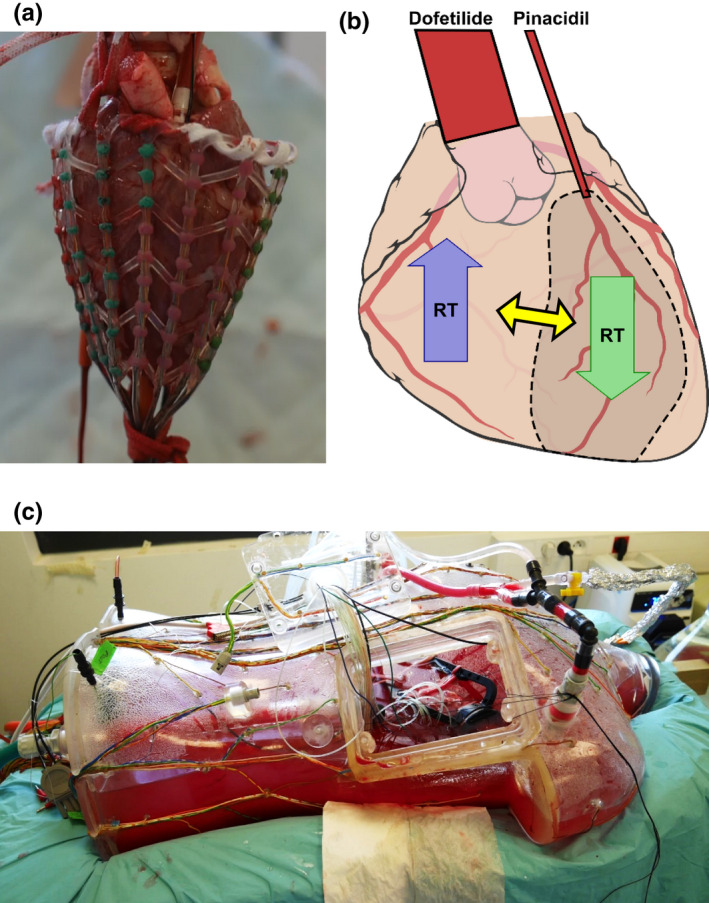
(a) Placement of the 108‐electrode epicardial sock around the heart, (b) schematic representation of creation of repolarization time (RT) gradient by regional infusion of RT prolonging drugs (dofetilide) and regional infusion of RT shortening drugs (pinacidil), (c) the heart was placed inside a human torso‐shaped tank with body‐surface electrodes (in this figure only halfway filled for illustration purposes).

Langendorff isolated heart experiments have previously been described in Bear et al. (2019, 2021); drug infusion and RT gradient determination have previously been described in Cluitmans et al. (2021). A brief summary is given here, and a more thorough description is given in the Appendix. In short, hearts of male pigs (*n* = 4; weight 33–41 kg) were explanted and connected to a Langendorff perfusion setup (Bear et al., 2019, 2021). The heart was paced from the right atrium (cycle length 400 or 450 ms). The left anterior descending artery (LAD) was cannulated and infused separately. Epicardial potentials were recorded with a 108‐electrode sock wrapped around the ventricles (Figure 1a).

Hearts were placed inside a human‐shaped torso tank (Figure 1c) equipped with 256 body‐surface ECG electrodes. Nine electrodes were selected to most accurately represent the 12‐lead ECG as measured in humans. Epicardial and body‐surface potentials were recorded simultaneously. From the measured unipolar electrograms, activation and repolarization times (ATs and RTs) were determined as the maximum downward slope during activation and the maximum upward slope during repolarization, relative to the QRS‐onset on the ECG.

A drug infusion protocol was used to create RT differences as follows: baseline (no drugs), dofetilide at 125 nM (Dof_half_), and 250 nM (Dof_full_) in the aorta‐perfused region (i.e., everywhere except LAD), and additional infusion of pinacidil at 17.5 μM (Dof_full_ + Pin_half_) and 35 μM (Dof_full_ + Pin_full_) in the LAD, followed by washout. This resulted in regions with pronounced RT prolongation (in the non‐LAD perfusion beds) and RT shortening (in the LAD perfusion bed) with steep gradients at their border (Figure 1b). Not all phases were implemented for all experiments. In heart 2, the dofetilide was not infused, and after full concentration of pinacidil, the heart went into VF. Defibrillation was not successful. Heart 4 went into refractory VF after infusion of dofetilide and half concentration pinacidil.

### 2.2 Repolarization gradient

An epicardial RT map was made from sock RTs (per electrode defined as the median RT of all electrodes within a radius of 20 mm) (Cluitmans et al., 2021). The local RT gradient (RTG, ms/cm) on each electrode position was defined as the largest local gradient steepness (RT difference divided by internode distance) within a 20‐mm distance. The 95th percentile value of all local RTGs was attributed to each condition. RT dispersion (RTD, in ms) was defined as the 95th percentile RT minus the 5th percentile RT.

### 2.3 T‐wave markers

**FIGURE 2:**
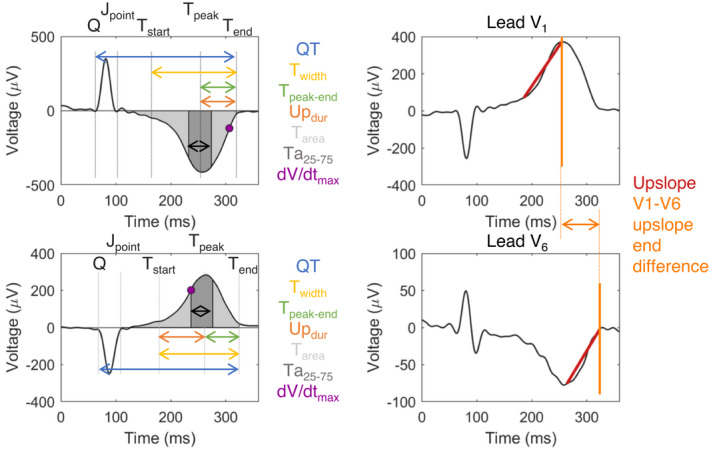
Illustration of the T‐wave parameters. The color of the mentioned parameter corresponds with the color of the arrow used.

ECG time indices were manually determined from the 12‐lead body‐surface ECG, filtered with linear offset removal, a bandpass 0.5–125 Hz filter and a 50 Hz notch filter. From the 12‐lead body‐surface ECG, several T‐wave parameters were manually determined (Table 1). QRS_start_ was determined as the time of the onset of QRS, J_point_ as time of end QRS, T_start_ as start of T wave using the inflection point at the onset of the T wave, T_peak_ as the time of maximum or minimum height of T wave, T_end_ as the end of the T wave (tangent method), see Figure 2. Leads with no distinguishable time indices (e.g., no T_peak_ due to biphasic T wave or flat T wave) were excluded from analysis. With these time indices, several composite T‐wave parameters were determined (Table 1). For the temporal, area(‐derived), and morphology(‐derived) metrics, both the mean and the maximum of all leads were determined, as well as the dispersion (max–min value in all 12 leads). Since all hearts were paced at a constant cycle length, we did not apply heart rate correction.

**TABLE 1:** Description of calculated T‐wave parameters

Temporal metrics	
QT_time_	Time between QRS_start_ and T_end_ (ms)
JT_time_	Time between J_point_ and T_end_ (ms)
T_peak–end_	Time between T_peak_ and T_end_ (ms)
T_width_	Time between T_start_ and T_end_ (ms)
TQ_ratio_	T_peak–end_ divided by QT_time_
dV/dt_max_	Time of maximum upward slope of T wave (ms)
Area‐derived metrics	
T_area_	Area under the T wave, from J_point_ to T_end_ (mV ms)
Ta_25–75_	Time difference between T‐wave area of 25% and 75% of maximum area (ms)
Morphology(‐derived) metrics	
T_amp_	Maximum amplitude of T wave (mV)
Upslope	Slope of the rising part of the T wave (mV/ms)
Upslope duration	Duration of rising part of the T wave (ms)
Other parameters	
V1–V_6_ upslope end difference	Time difference between the end of the upslope of lead V_1_ and the end of the upslope of lead V_6_

### 2.4 Statistics and data analysis

Each heart served as its own control. For each parameter, Pearson's linear correlation coefficient *R* between the value of the parameter and the steepness of RTG is determined. A *p*‐value smaller than .05 was considered significant.

## 3 RESULTS

### 3.1 Creation of repolarization gradient

**FIGURE 3:**
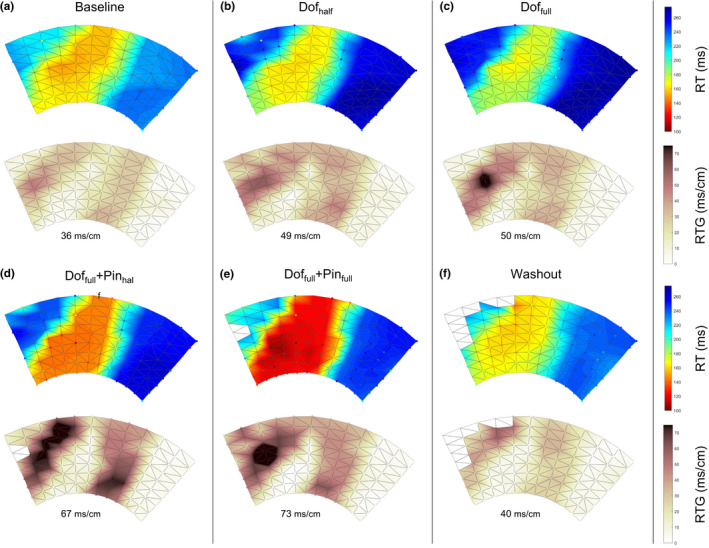
Measured repolarization times (RTs, top) on the 108‐electrode epicardial sock and corresponding RT gradients (RTGs, bottom) for heart 1 in all six different phases of drug infusion. Values below indicate the maximum RTG for each RT map.

**FIGURE 4:**
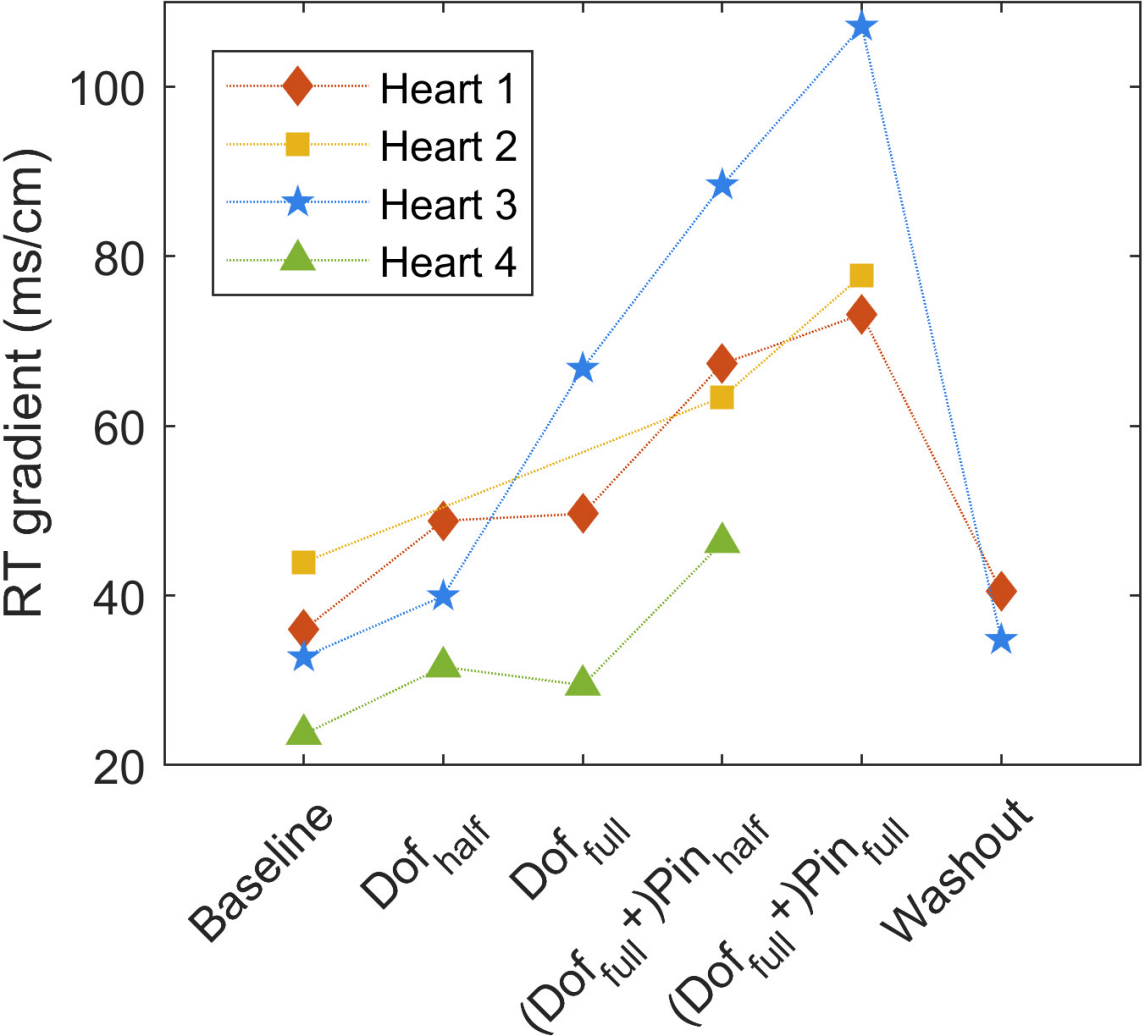
Maximum repolarization time gradient per phase for all different hearts

Figure 3 shows an example of the RTs and corresponding RTGs during six different phases of one experiment, displayed on the unfolded epicardial electrode sock. The local RTs and total RT dispersion increase with the infusion of the drugs. Infusion of dofetilide in the aorta causes prolonged repolarization in the non‐LAD area (b & c). Simultaneous LAD‐infusion of pinacidil shortens repolarization in LAD area (d), in a concentration‐dependent fashion (e). The corresponding RTG maps highlight the presence of steep gradients at the border of the LAD region. After washout of the drugs, the repolarization times approximately return to baseline values (f). In all experiments, the RTG steepness follows this pattern (Figure 4). RT dispersion (range of minimum RT to maximum RT) and RT gradients (local steepness of heterogeneity) correlate (Figure S1).

**FIGURE 5:**
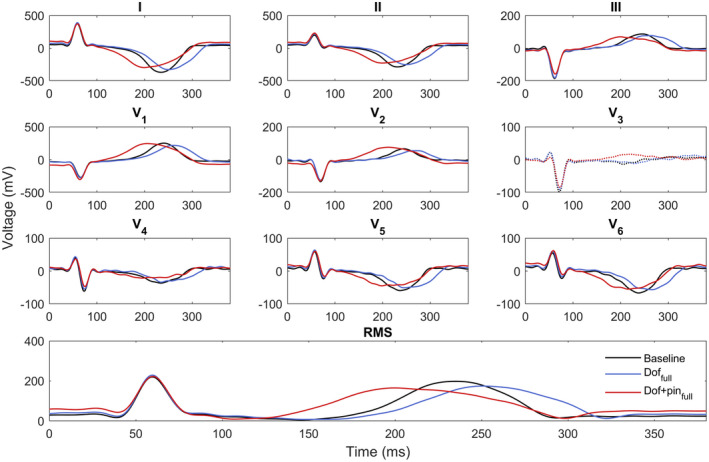
Example of ECGs in heart 1 in different stages of drug infusion. In this experiment, the ECGs of lead V_3_ are excluded due to low voltage T waves.

Figure 5 shows an example of the ECG from the same experiment as Figure 3. Upon drug infusion, significant T‐wave changes can be observed in all ECG leads. With dofetilide, a prolongation of the QT interval and a widening of the T wave is seen. Additional infusion of pinacidil advanced the upslope of the T wave, while the end of the T wave returned to baseline values. Note that the normalization of the end of the T wave implies that a normal QT occurred when RT gradients were steepest. In this experiment, lead V_3_ was excluded due to low voltage T waves. The same T‐wave changes can be observed in the RMS signal (obtained from 12‐lead ECG, lower panel).

### 3.2 T‐wave metrics versus repolarization gradient

**FIGURE 6:**
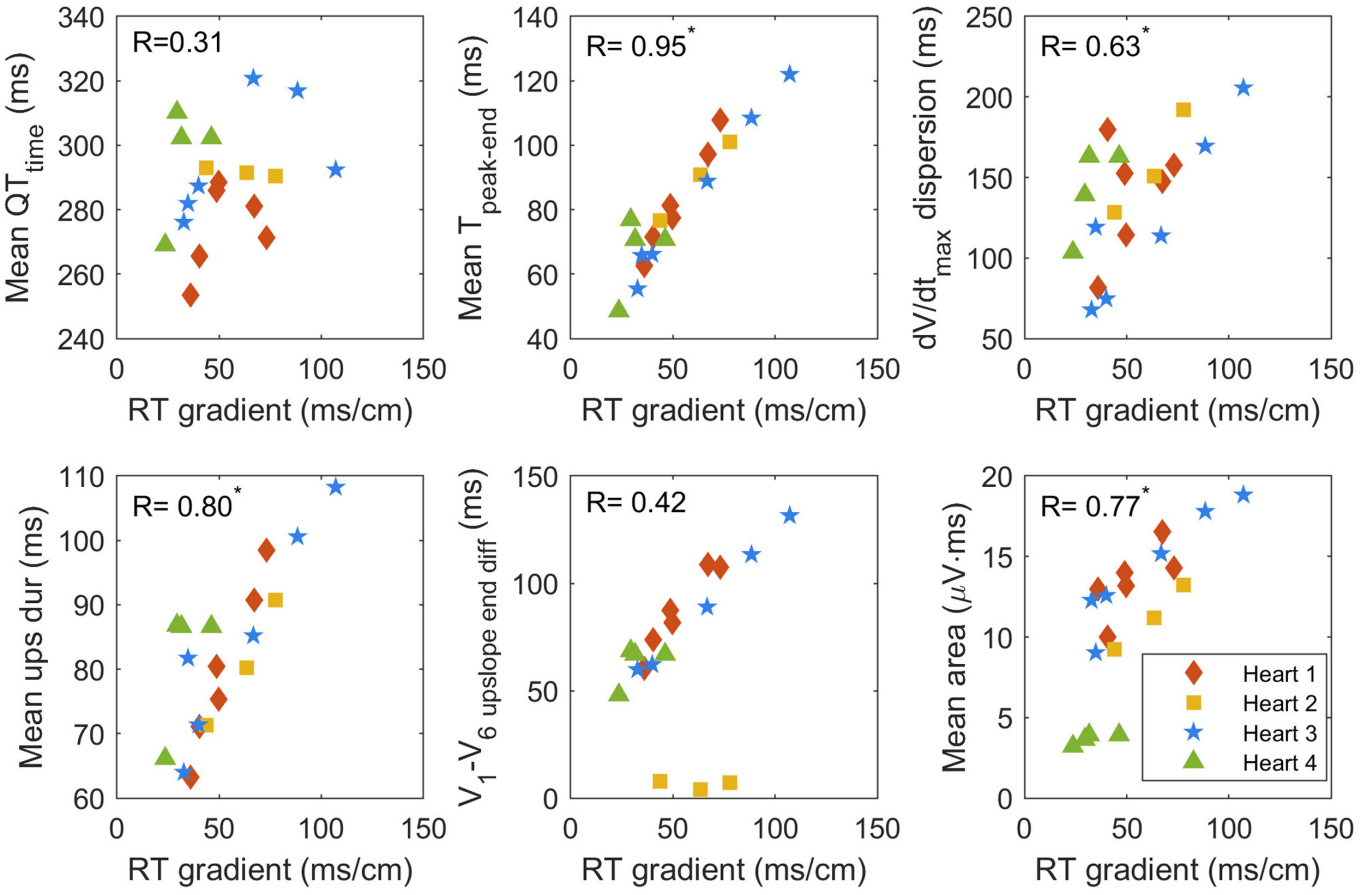
Correlation plots of repolarization gradient versus selected ECG T‐wave parameters. Correlation coefficient *R* is mentioned in the figure, with * indicating a *p*‐value of <.05.

The value of the RTG is plotted against T‐wave parameters in Figure 6. The correlations with T‐wave parameters (and their mean, maximum, and dispersion) and their significance are given in Table 2. There is a poor correlation between mean, maximum, or dispersion of QT_time_ and RT gradient. The temporal T‐wave parameters T_peak–end_, T_width,_ and TQ_ratio_ show a significant and high correlation with RT gradient. The mean value of these parameters had a higher correlation than its maximum or dispersion values. The dV/dt_max_ only shows a high correlation for dispersion, albeit lower than aforementioned temporal T‐wave parameters. T_area_ shows a similar significant correlation for mean, max, and dispersion. Upslope duration shows a significant correlation in particular for mean values. V_1_–V_6_ upslope end difference was not significantly correlated with steepness of RT gradient in this study, although the correlation plot in Figure 6 shows that this is mainly due to an absence of correlation with RT gradient in heart 2. All parameters have also been investigated for correlation with RT dispersion, and results are similar to those calculated with RT gradients (Table S1).

**TABLE 2:** Correlation value (Pearson's *R*) between the maximum epicardial RT gradient and the mean, max, and dispersion of selected T‐wave parameters of the body‐surface ECG leads

	Mean	Max	Dispersion
	*R*	*R*	*R*
Temporal metrics			
QT_time_	.31	.23	−.18
JT_time_	.28	.22	−.11
T_peak–end_	.95*	.93*	.33
T_width_	.91*	.74*	.69*
TQ_ratio_	.92*	.91*	.30
dV/dt_max_	.01	.26	.63*
Area (‐derived) metrics			
T_area_	.77*	.69*	.68*
Ta_25–75_	−.05	−.23	−.43
Morphology (‐derived) metrics			
T_amp_	.13	.27	.30
Upslope duration	.80*	.47*	.02
Upslope	.20	.21	.10

Other parameters	R
V_1_–V_6_ upslope end diff	.42

* *p* < .05.

## 4 DISCUSSION

We have investigated various electrocardiographic parameters that are thought to give information about underlying repolarization patterns and RT gradients. The use of a torso tank with a submerged explanted heart with separate infusion beds enabled us to artificially create different levels of repolarization heterogeneity, allowing comparison of the change in T‐wave parameters within and between subjects. Our results show that both the interval between T_peak_ and T_end_ (T_peak–end_) and the duration of the T wave (T_width_) have a very high correlation with underlying drug‐induced repolarization gradients. Contrastingly, the clinically widely used parameters for repolarization QT_time_ and QT_dispersion_ did not correlate with the underlying RT gradient.

### 4.1 Parameter interpretation and previous studies

#### 4.1.1 QT_time_

The QT interval relates the latest moment of repolarization to the earliest moment of activation (at potentially different locations). Therefore, an increased QT_time_ may be due to APD changes, but may also reflect (regional) differences in activation time or conduction. Additionally, QT prolongation may occur without increasing RT gradients, for example, when all RTs are prolonged homogeneously. This theoretically limits the use of QT_time_ for assessing repolarization heterogeneity and may explain disparate results in predicting arrhythmia vulnerability (Anderson, 2006; Hondeghem, 2011). This is confirmed in our study. Although dispersion of QT has been suggested to reflect abnormal ventricular repolarization (Statters et al., 1994), it may be caused by the different projection of the T‐wave vector in different ECG leads (Lee et al., 1998). Our findings support the lack of relation between mean, max, or dispersion in QT_time_ and RT gradients. We therefore also investigated composite T‐wave parameters.

#### 4.1.2 T_peak–end_ and T_width_

T_peak–end_ has been a topic for discussion in relation to repolarization heterogeneity in recent literature (Malik et al., 2019). Studies report a relation between T_peak–end_ and arrhythmia risk in patients with heart failure (Rosenthal et al., 2015), ischemic heart disease (Shenthar et al., 2015) and BrS (Castro Hevia et al., 2006), although other studies contradict that prolonged T_peak–end_ interval is proarrhythmic (Michalek et al., 2020; O'Neal et al., 2017). We have previously shown that the onset of the electrocardiographic T wave occurs before first measured RT and that 75% of recording sites repolarized after T_peak_ (Meijborg et al., 2014). Combined, this suggests that T_peak–end_ reflects the majority of the repolarization phase and that T_peak–end_ is a measure of global repolarization heterogeneity (Opthof et al., 2007). Indeed, we found a significant correlation of both T_peak–end_ and T_width_ with RTG. Because the determination of T‐wave onset is often difficult, T_width_ is likely to be less accurate as a parameter for repolarization heterogeneity than T_peak–end_.

Srinivasan et al. did not observe a correlation between T_peak–end_ and dispersion of repolarization along any major anatomical axis (Srinivasan et al., 2019). However, dispersion of repolarization was assessed by contact catheter recordings during an invasive study, and only global left–right and apex‐base repolarization dispersion was studied, not pathologically increased RTGs. In our recent study in which an RT gradient was pharmacologically induced, the RTGs correlated well with increased arrhythmia vulnerability (Cluitmans et al., 2021).

#### 4.1.3 Amplitude/area

The amplitude and area of the T wave also have been considered as a marker for dispersion of repolarization or even as a prognostic risk marker for cardiovascular death (Arteyeva & Azarov, 2017). We did not find a significant correlation between T_amp_ and RTG. Mean T_amp_ is even constant within each heart during all phases of drug infusion, although it varies between hearts (Figure S2). This complies with the observation that ECG amplitude is sensitive to individual patient characteristics (Rautaharju et al., 1994).

Measuring the T‐wave area does not rely on precise determination of the start and end of the T wave and is not hampered by flat or biphasic T wave. T‐wave area, which combines morphology and time parameters, is expected to correlate to (local) dispersion of repolarization. Indeed, Punke et al. show a significant correlation between T‐wave area and dispersion of repolarization in isolated canine hearts (Punske et al., 1999). Additionally, if T_amp_ is independent of RTG, and T_width_ and T_peak–end_ are directly related to RTG as is apparent from our data, then T_area_ should relate to RTG as well. This is supported by Kentta et al., who showed that dispersion in T‐wave area is a predictor for SCD in the adult population (Kentta et al., 2018).

#### 4.1.4 TQ_ratio_

TQ_ratio_ is a predictor for SCD in patients with gene‐associated hypertrophic cardiomyopathy (Shimizu et al., 2002) and was associated with arrhythmia inducibility in patients with Brugada syndrome (BrS) (Letsas et al., 2010). However, a large retrospective study in patients with BrS did not show a difference in T_peak–end_ or TQ_ratio_ between asymptomatic patients and those with syncope and malignant arrhythmias (Mugnai et al., 2017). While the literature is equivocal, we show that both T_peak–end_ and TQ_ratio_ show a similarly high correlation with repolarization gradient. TQ_ratio_ is supposed to be independent of heart rate. Since in our experiments, the heart rate was kept constant, we could not investigate this substantial benefit in clinical situations to determine cut‐off values for risk stratifications that are independent of patient's heart rate.

#### 4.1.5 dV/dt max dispersion

If the ECG reflects a local unipolar electrogram, the dV/dt_max_ of the T wave likely provides information about regional repolarization (Orini et al., 2018). Indeed, it has been shown that RV repolarization occurred on the upslope of leads V_1_, V_2_, and V_3_, while LV repolarization occurred on the upslope of leads V_6_ and I (Srinivasan et al., 2019). We indeed found a correlation between dV/dt max dispersion and repolarization gradient (albeit not as strong as for T_peak–end_). In addition, this parameter may be more sensitive to the *orientation* of RT gradients than time‐dependent metrics.

#### 4.1.6 Upslope metrics

The design of the upslope metric is based on the observation that the polarity of the T wave on the precordial leads reflects the polarity of the unipolar signal recorded on the nearby myocardium (Srinivasan et al., 2019). Per definition, the upslope duration equals T_start_–T_peak_ for positive T waves and T_peak_–T_end_ for negative T waves. In the unipolar electrogram, the time of repolarization is determined by time of dV/dt_max_ of the T wave, irrespective of its polarity (Coronel et al., 2006). In our study, the upslope duration was calculated over all leads and correlated significantly with RTG. Including precordial leads only cause a small decrease in the correlation parameters (data not shown). Given that the upslope duration and T_peak–end_ are the same for negative T waves, the lower correlation of upslope duration suggests that this metric likely depends on its correlation with T_peak–end_, and thus on the presence of negative T waves. If we excluded negative T waves (*n* = 89 in the total selection), the correlation values decreased, while if we excluded positive T waves (*n* = 109 in the total selection) from analyses the correlation values increased. This indeed suggests that T_peak–end_ is a more predictive parameter for RTG than upslope duration.

The difference between V_1_ and V_6_ upslope end, introduced by Srinivasan et al. (2019), showed a correlation with right‐to‐left dispersion in their dataset, but did not show a significant correlation with repolarization gradient in our data. This metric depends on the polarity of the T waves (Figure 6: heart 2 has positive T waves in V_1_ and V_6_; thus, V_1_–V_6_ upslope end difference equals a difference between T_peak_ in both leads, whereas this equals a difference between a T_peak_ and a T_end_ in other hearts that have a positive T wave in lead V_1_ and a negative T wave in lead V_6_). This indicates that this metric is sensitive to orientation or location of the RT gradient and suggests that combined temporal metrics such as T_peak–end_ would be a more reliable measure of repolarization dispersion.

### 4.2 Limitations

The torso tank is an accepted technique to study the relation between local changes in cardiac electrophysiology to the surface ECG (Lux, 2002), with exclusion of confounding factors (the autonomic nervous system and hemodynamics). However, even in a tank shaped like a human torso, the use of a pig heart prevents the exact replication of a human 12‐lead ECG. In pig hearts, the QRS complex and T wave are typically discordant, while in human, they are generally concordant. Nevertheless, the changes in T wave due to increased RT gradients should be comparable between pigs and humans. Studies that employed repolarization vectorcardiography in humans support extension of our mechanistic findings to human application, and may help in assessing repolarization gradients in more detail under clinical conditions (Perez‐Alday et al., 2019; van Dam et al., 2021).

The local infusion of pharmacologic agents allowed us to reliably create RTGs of varying steepness and to investigate its influence on the body‐surface ECG within the same individual in the absence of confounding factors that influence repolarization. As a result of this approach, the RTGs investigated in this study are steep and are present in a large region of the heart. RT gradients in patients at risk for cardiac arrest may be more subtle, although studies describing detailed repolarization patterns in both healthy and diseased hearts are scarce. It has been shown that increased RTGs lead to increased arrhythmia vulnerability for a single extra‐stimulus (Cluitmans et al., 2021). In addition, the steepness of RT gradients as observed with non‐invasive mapping is related to arrhythmia occurrence in humans (Cluitmans et al., 2021; Zhang et al., 2017).

The selected 12‐lead T‐wave markers were compared with regional measures of epicardial RT gradients as a gold standard. We have no information about endocardial RTs, and therefore also not about transmural RT gradients. However, we previously demonstrated that transmural dispersion of repolarization in the human and pig heart is negligible (Meijborg et al., 2014; Opthof et al., 2016). In addition, a study with a similar dataset showed that local drug infusion through the major arteries did not lead to changes in dispersion of transmural RTs (Meo et al., 2020).

## 5 CONCLUSION

Twelve‐lead ECG T‐wave parameters T_peak–end_, T_width,_ and TQ_ratio_ show a good quantitative correlation with underlying repolarization heterogeneity that is lacking for standard clinical metrics such as QT_time_. In our drug‐induced RTG model, repolarization heterogeneity is better characterized by composite parameters highlighting multiple moments of *local* repolarization (T_width_, T_peak–end_, and T_area_) than by parameters reflecting the single time point of latest *global* repolarization (QT_time_ and QT_dispersion_). Our data suggest that T_peak–end_ or T_width_ contains information about both repolarization duration and heterogeneity, contrary to clinically used parameters QT_time_ and QT_dispersion_. In addition, TQ_ratio_ is a potentially valuable parameter for repolarization heterogeneity as it is independent on a patient's heart rate. When studying the role of repolarization in human arrhythmogenesis, the composite temporal T‐wave metrics may thus provide better insights in arrhythmia susceptibility than traditional metrics.

### AUTHOR CONTRIBUTION

JW drafted the manuscript, LB and MC performed the animal experiments, JW, LB, VM and MC performed data analysis, MC and RC supervised this work. All authors contributed to study design, and all authors critically revised the article and accepted its final version.

## FUNDING INFORMATION

This work was supported by the Leducq Foundation [RHYTHM transatlantic network, grant number 16CVD02], and the French National Research Agency (ANR‐10‐IAHU04‐LIRYC). Matthijs Cluitmans is supported by a Veni grant from The Netherlands Organization for Scientific Research (TTW 16772).

## CONFLICT OF INTEREST

MC is part‐timely employed by Philips Research, Eindhoven, The Netherlands.

## ETHICAL STATEMENT

All experiments were approved by the local ethical committee of Bordeaux CEEA50, and animal handling was in accordance with the European Directive for the Protection of Vertebrate Animals Used for Experimental and Other Scientific Purposes (European Union Directive 86/609/EEC).

## Supporting information


Appendix S1

## Data Availability

The data that support the findings of this study are available from the corresponding author upon reasonable request.
