# Supplementary material for: Steep repolarization time gradients in pig hearts cause distinct changes in composite electrocardiographic T‐wave parameters
Source: Ann Noninvasive Electrocardiol. 2022 Aug 19;27(6):e12994. doi: 10.1111/anec.12994 (PMC9674780; doi:10.1111/anec.12994)
Supplement: Supplementary file 1 — Appendix S1 [file ANEC-27-e12994-s001.docx]

# Supplementary Material

## ***Supplemental methods***

In this supplemental methods section we describe both the experimental and analytical methodology in more detail.

## *Experimental protocol*

All experiments were approved by the institutional review committee for experiments on animals, and animal handling was in accordance with the European Directive for the Protection of Vertebrate Animals Used for Experimental and Other Scientific Purposes (European Union Directive 86/609/EEC). Male pigs (n=4; weight 33 – 41 kg, from the same strain and raised under the same conditions) were premedicated, anaesthetized, intubated and artificially ventilated. Heparin (5000IU) was injected intravenously. The heart was exposed by a midsternal thoracotomy. Blood was collected and ventricular fibrillation (VF) was electrically induced. The heart was isolated and immersed into an ice-cold Tyrode’s solution (mM: 155.5 Na, 4.7 K, 1.45 Ca, 0.6 Mg, 136.5 Cl, 27.0 HCO3, 0.4 PO4, 11.1 glucose, pH = 7.35–7.45). The aorta was cannulated, connected to a recirculatory Langendorff perfusion system and retrogradely perfused with a 1:1 blood-Tyrode’s mixture kept at 37.5ºC. The heart was defibrillated and paced from the right atrium to provide a baseline paced rhythm through the native ventricular conduction system to mimic sinus rhythm. This was performed at the longest cycle length providing continuous capture, typically 450 or 500 ms. The left anterior descending artery (LAD) was briefly occluded to define its perfusion domain (usually the lower anterior part of the left ventricle (LV), apex and a small anterior part of the right ventricle (RV)) and subsequently cannulated. Epicardial potentials were recorded with a 108-electrode sock wrapped around the ventricles (Figure 1A).

A drug infusion protocol was used to create repolarization time (RT) differences, as previously described by Cluitmans et al. (1): Dofetilide target concentration was taken from literature as 100 nM in a no-blood preparation (2). To compensate for the use of blood, we corrected for known plasma binding of 60 to 70% of dofetilide (3) by multiplying this concentration by 2.5, resulting in a 250 nM target concentration. Pinacidil target concentration from literature was 20 µM in a no-blood preparation (4) and multiplied by 1.67 to compensate for its 40% plasma binding (5), yielding a final target concentration of 35 µM. Both drugs were also infused at half concentrations (125 nM for dofetilide and 17.5 µM for pinacidil) to test gradual increases. Each drug was infused for at least 15 min before taking recordings. The drugs were given in the following order: baseline (no drugs), dofetilide at 125 nM (“Dof_half_”) and 250 nM (“Dof_full_”) in the aorta-perfused region (thus everywhere except LAD), and, on top of Dof_full_, an additional infusion of pinacidil at 17.5 µM (“Dof_full_ + Pin_half_”) and 35 µM (“Dof_full_ + Pin_full_”) in the LAD, followed by washout. This resulted in regions with pronounced RT prolongation (in the non-LAD region) and RT shortening (in the LAD region) with increasingly steep gradients at their border.

Hearts were placed inside a human-shaped torso-tank (Figure 1C) equipped with 256 body surface ECG electrodes. Unfortunately, due to limited room inside the tank, the heart could not always be placed exactly at the position and orientation it would have inside a patient’s torso. Therefore, we selected those electrodes for the 12-lead ECG that best represented the position of the heart in patient’s torso (e.g., electrodes V_1_ to V_6_ are chosen to reflect right ventricle going to left ventricle). These same electrodes were used for the remainder of each experiment, so all observed ECG changes were the direct result of drug perfusion and their RT effects.

## *Signal analysis*

Both epicardial potentials and body surface ECGs were recorded simultaneously using a BioSemi system (BioSemi, the Netherlands, 2048 Hz, bandwidth (-3dB) DC -400 Hz, 24 bit dynamic range, 122.07 nV LSB, total noise 0.5 µV). Per distinct experimental phase (e.g., at each change in drug infusion concentration) we analyzed a single -representative- beat, and correlated the T-wave markers with the underlying repolarization gradient determined from this same beat. Alternans was not observed. No signal averaging was performed.

From the measured unipolar electrograms, activation times (ATs) were determined as the maximum downward slope of the QRS complex and RTs were determined as the maximum upward slope of the T-wave (6, 7) with respect to the onset of QRS on the ECG. This was performed offline using custom-made software based on Matlab with automatic dV/dt annotations which were manually checked. The resulting repolarization map was then filtered with a 20-mm spatial filter; the RT on one electrode location was determined by taking the median RT of all electrodes within a radius of 20 mm. From this map, the local RT gradient (in ms/cm) was determined for each electrode location by taking the largest RT gradient (RT difference divided by interelectrode distance) with its neighbouring electrodes within a radius of 20 mm. The choice for the 20 mm spatial filter and 20 mm radius for RTG determination was based on data from an earlier study (1) and the typical inter-electrode distance of the sock (ranging from 9-17 mm). Then, an overall RTG was determined by taking the 95^th^ percentile of all local RTGs, therefore taking the highest RTG but excluding outliers.

The 12-lead ECG measured from the torso tank was filtered with linear offset removal, a bandpass -.5-125 Hz filter and a 50 Hz notch filter. During each drug infusion phase, one beat was selected. ECG leads with a flat T-wave were excluded from analysis, because measurement of T-wave peaks was impossible in these leads.

## *Model limitations*

Torso models have been used to better understand the relation between the ECG and local activation and repolarization parameters since the 1950's, and have led to significant increment of basic knowledge on the subject (8-10). The torso model has the advantage over the in situ heart that less readily controlled parameters such as ventricular pressure and work, and the influence of the autonomic nervous system can be excluded. We therefore used the isolated heart model in the torso tank to apply controlled changes of repolarization without altering activation. In addition, the torso tank allows us to compare different (artificially created) RT gradients within the same subject, which is not possible in human clinical studies. The ECG and patterns in activation and repolarization show similarities between pigs and humans (11), which facilitates translatability to human.

One major difference of the isolated heart model with the human torso is the presence of other tissue, e.g. lungs, bones and body fat. These structures have a lower conductivity than the conductive fluid used to fill the tank. Therefore, local effects of RT changes may be attenuated by the presence of these tissues, although this primarily refers to the amplitude of the signal, not to its morphology or timing. In addition, it has been shown that relative to a homogeneous torso, torso inhomogeneities only have a minor effect on body surface potentials (12).

## ***Supplemental tables & figures***


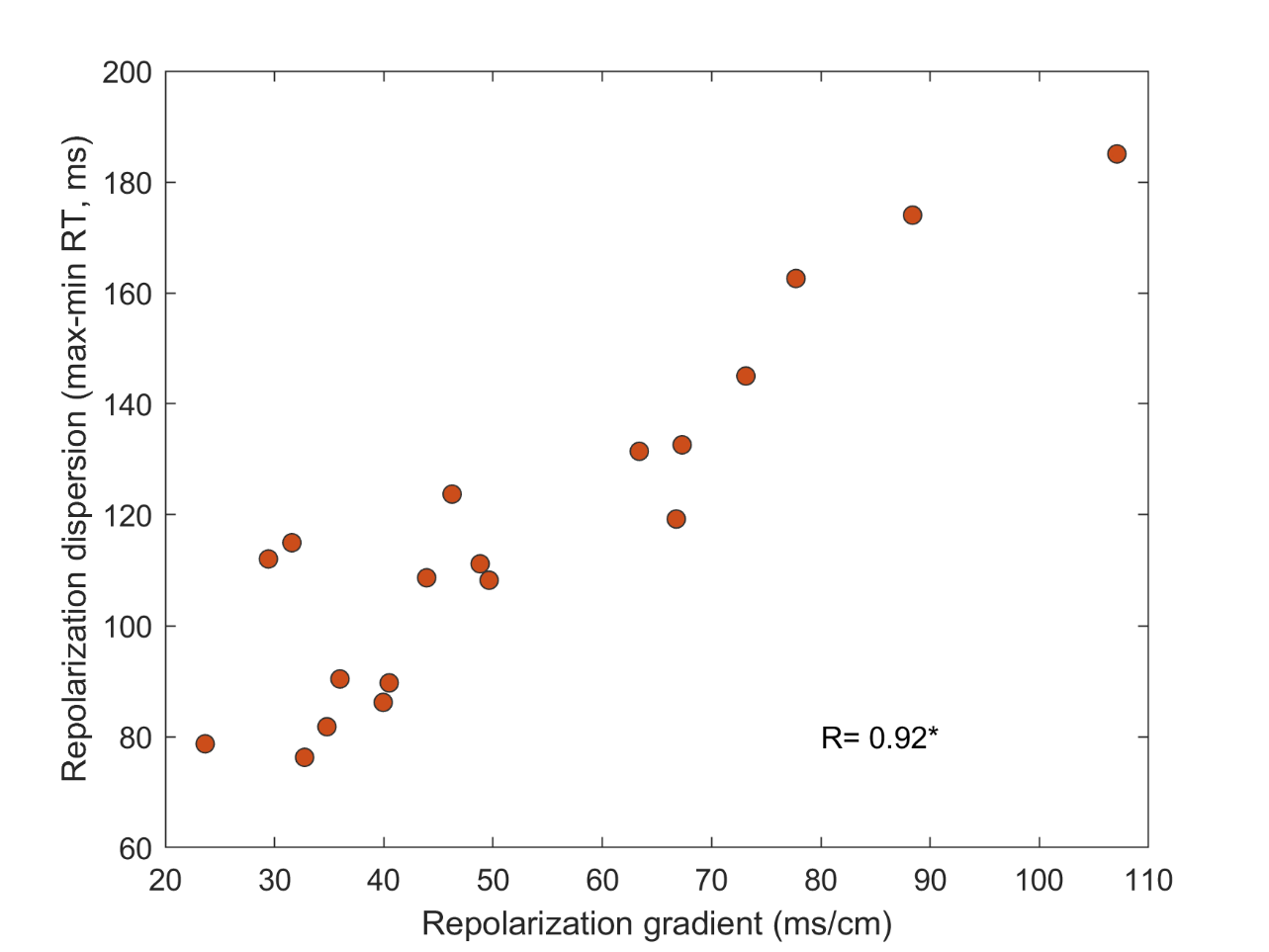


Supplementary Figure 1: Repolarization dispersion (range of minimum RT to maximum RT, in ms) versus repolarization gradients (local steepness of heterogeneity, in ms/cm), showing a statistically significant correlation between the two (R=0.92).

### Correlations for all parameters with the global RT dispersion instead of local RT gradient

Supplementary Table 1: Correlation value between the epicardial RT dispersion and the mean, max and dispersion of selected T-wave parameters of the body-surface ECG leads. * indicates p<0.05.

| ***Temporal metrics*** | **Mean** | **Max** | **Dispersion** |
| --- | --- | --- | --- |
|  | **R** | **R** | **R** |
| QT_time_ | 0.45 | 0.41 | 0.05 |
| JT_time_ | 0.36 | 0.31 | 0.06 |
| T_peak-end_ | 0.94* | 0.96* | 0.53* |
| T_width_ | 0.96* | 0.86* | 0.83* |
| TQ_ratio_ | 0.88* | 0.93* | 0.50* |
| dV/dt_max_ | -0.31 | -0.03 | 0.77* |
|  | | | |
| ***Area (-derived) metrics*** | **Mean** | **Max** | **Dispersion** |
|  | **R** | **R** | **R** |
| T_area_ | 0.52* | 0.46 | 0.46 |
| Ta_25-75_ | 0.29 | 0.10 | -0.11 |
|  | | | |
| ***Morphology (-derived) metrics*** | **Mean** | **Max** | **Dispersion** |
|  | **R** | **R** | **R** |
| T_amp_ | 0.07 | -0.01 | -0.01 |
| Upslope duration | 0.89* | 0.64* | 0.21 |
| Upslope | 0.12 | -0.07 | -0.20 |
|  | | | |
| ***Other parameters*** | **R** |  | |
| V1-V6 upslope end diff | 0.34 |  |  |


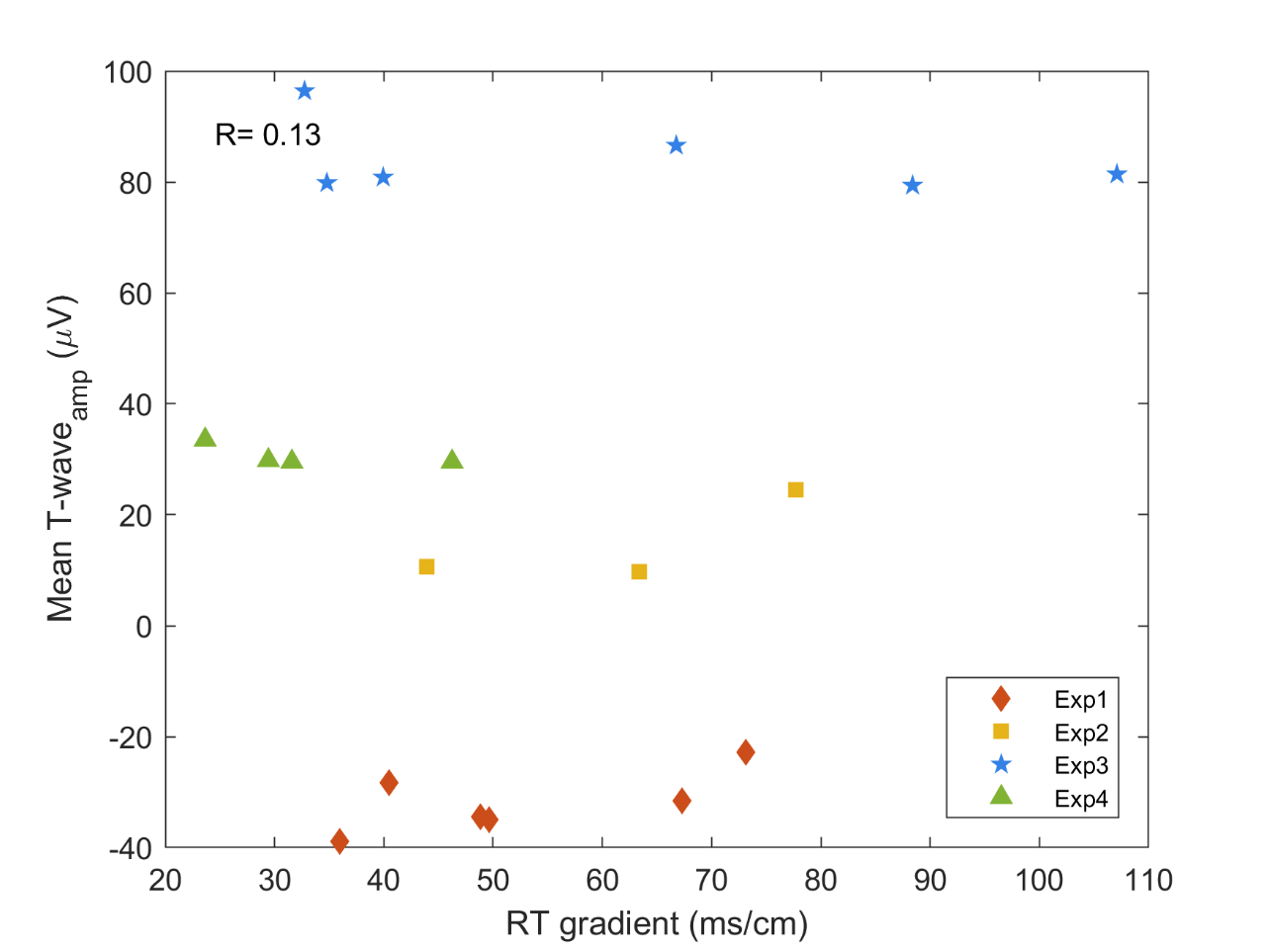


Supplementary Figure 2: Mean T-wave amplitude versus RT gradient, showing no correlation between the two (R=0.13).

## ***References***

1. Cluitmans MJM, Bear LR, Nguyên UC, van Rees B, Bekke RMA, Mihl C, et al. Noninvasive detection of spatiotemporal activation-repolarization interactions that prime idiopathic ventricular fibrillation. Sci Transl Med. 2021;13(620).

2. Laursen M, Grunnet M, Olesen SP, Jespersen T, Mow T. Keeping the rhythm -- pro-arrhythmic investigations in isolated Gottingen minipig hearts. J Pharmacol Toxicol Methods. 2011;64(2):134-44.

3. National Center for Biotechnology Information: Dofetilide, CID=71329PubChem; [Available from: <https://pubchem.ncbi.nlm.nhi.gov/compound/Doufetilide#section=Absorption-Distribution-and-Excretion>.

4. Coronel R, Wilms-Schopman FJ, Opthof T, Janse MJ. Dispersion of repolarization and arrhythmogenesis. Heart Rhythm. 2009;6(4):537-43.

5. Ward JW, McBurney A, Farrow PR, Sharp P. Pharmacokinetics and hypotensive effect in healthy volunteers of pinacidil, a new potent vasodilator. Eur J Clin Pharmacol. 1984;26(5):603-8.

6. Coronel R, de Bakker JM, Wilms-Schopman FJ, Opthof T, Linnenbank AC, Belterman CN, et al. Monophasic action potentials and activation recovery intervals as measures of ventricular action potential duration: experimental evidence to resolve some controversies. Heart Rhythm. 2006;3(9):1043-50.

7. Haws CW, Lux RL. Correlation between in vivo transmembrane action potential durations and activation-recovery intervals from electrograms. Effects of interventions that alter repolarization time. Circulation. 1990;81(1):281-8.

8. Messinger-Rapport BJ, Rudy Y. Noninvasive recovery of epicardial potentials in a realistic heart-torso geometry. Normal sinus rhythm. Circ Res. 1990;66(4):1023-39.

9. Scher AM. Studies of the electrical activity of the ventricles and the origin of the QRS complex. Acta Cardiol. 1995;50(6):429-65.

10. Toyoshima H. Correlations between the spread of ventricular activation and map patterns of measured and difference maps. Am Heart J. 1976;92(2):183-92.

11. Meijborg VM, Conrath CE, Opthof T, Belterman CN, de Bakker JM, Coronel R. Electrocardiographic T wave and its relation with ventricular repolarization along major anatomical axes. Circ Arrhythm Electrophysiol. 2014;7(3):524-31.

12. Ramanathan C, Rudy Y. Electrocardiographic imaging: I. Effect of torso inhomogeneities on body surface electrocardiographic potentials. J Cardiovasc Electrophysiol. 2001;12(2):229-40.
